# The helicase, DDX3X, interacts with poly(A)-binding protein 1 (PABP1) and caprin-1 at the leading edge of migrating fibroblasts and is required for efficient cell spreading

**DOI:** 10.1042/BCJ20170354

**Published:** 2017-08-31

**Authors:** Alice C. Copsey, Simon Cooper, Robert Parker, Ella Lineham, Cuzack Lapworth, Deema Jallad, Steve Sweet, Simon J. Morley

**Affiliations:** 1 Department of Biochemistry, School of Life Sciences, University of Sussex, Brighton BN1 6SE, U.K.; 2 Faculty of Medicine, National Heart & Lung Institute, St Mary's Campus, Imperial College London, London W2 1NY, U.K.; 3 Genome Damage and Stability Centre, School of Life Sciences, University of Sussex, Brighton BN1 9RQ, U.K.

**Keywords:** cell migration, DDX3X, translation, translation factors

## Abstract

DDX3X, a helicase, can interact directly with mRNA and translation initiation factors, regulating the selective translation of mRNAs that contain a structured 5′ untranslated region. This activity modulates the expression of mRNAs controlling cell cycle progression and mRNAs regulating actin dynamics, contributing to cell adhesion and motility. Previously, we have shown that ribosomes and translation initiation factors localise to the leading edge of migrating fibroblasts in loci enriched with actively translating ribosomes, thereby promoting steady-state levels of ArpC2 and Rac1 proteins at the leading edge of cells during spreading. As DDX3X can regulate Rac1 levels, cell motility and metastasis, we have examined DDX3X protein interactions and localisation using many complementary approaches. We now show that DDX3X can physically interact and co-localise with poly(A)-binding protein 1 and caprin-1 at the leading edge of spreading cells. Furthermore, as depletion of DDX3X leads to decreased cell motility, this provides a functional link between DDX3X, caprin-1 and initiation factors at the leading edge of migrating cells to promote cell migration and spreading.

## Introduction

DEAD-box proteins are ATP-dependent RNA-binding proteins that remodel RNA structures and RNA–protein complexes [1]. DDX3X, as a member of the DEAD/H box helicase family, participates in several steps of gene expression including translation [2] and also has a role in antiviral innate immunity [3–5]. It is a member of the Ded1/DDX3 helicase subfamily, along with the *Saccharomyces cerevisiae* orthologue, Ded1p [2,6–8]. The N-terminal tail of DDX3X contains an eIF4E-binding motif [9], whereas the C-terminal tail contains conserved sequences of unknown function that are essential for oligomerisation. Ded1p is an essential protein that acts both as a repressor of translation initiation through its ability to interact with other translation initiation factors and as an activator via its ATP-dependent activity [2,6,8]. DDX3X can function in cell signalling [10] and is frequently mutated in cancers such as chronic lymphocytic leukaemia [11], lymphoma [12], head and neck squamous cell carcinoma [13], breast [14] and lung cancer [15]. It is also one of the most frequently mutated genes in medulloblastoma [16–20] where documented mutations inactivate DDX3X RNA helicase activity [21].

DDX3X can interact directly with mRNA regulating the selective translation of mRNAs that contain a structured 5′-untranslated region (5′-UTR) [3,22]. It regulates the expression of cyclin E1 mRNA [23] and modulates efficient expression of Rac1, thereby regulating actin dynamics [24] and contributing to cell adhesion and motility [25]. DDX3X is known to contribute to the formation of cytoplasmic stress granules [26], which sequester mRNAs in response to exogenous or endogenous stress and, with the exception of some stress-related mRNAs, halts their translation [27,28]. It can inhibit viral mRNA translation by binding to eIF4E–viral mRNP complexes, trapping them in a translationally inactive state and thereby sequestering the eIF4E–viral mRNPs into stress granules [29].

Another mRNA-binding protein present in both polysomal and translationally silent mRNPs is the proliferation-regulated protein, caprin-1 [30–32]. Caprin-1 can be localised to the leading edge of cells [33], but as with DDX3X, it can relocalise to stress granules containing stalled mRNAs. The carboxy-terminal region of caprin-1 selectively binds c-myc and cyclin D2 mRNAs using RGG domains [33] and interacts directly with RasGap SH3 domain-binding protein-1 (G3BP-1) to promote stress granule formation [34].

In addition to eIF4E, mammalian DDX3X has been reported to interact with eIF3 [35], poly(A)-binding protein 1 (PABP1) [26] and eIF4GI [3]. PABP1 binds to both the mRNA poly(A) tail and eIF4GI governing the stability and translation of mRNA [36]. Although its exact role is unknown, DDX3X is believed to facilitate 40S ribosome scanning of the 5′-UTR of mRNAs containing secondary structure and promote 80S ribosome assembly [3,4,26,35]. It can unwind secondary structure proximal to the 5′-cap and substitute for eIF4E to form a DDX3X/PABP1/eIF4GI complex on HIV genomic mRNA [3,4].

Previously, we have shown that initiation factors and PABP1 [37] localise to the leading edge of cells in loci enriched with actively translating ribosomes [38]. PABP1 generally shows a diffuse cytoplasmic distribution, actively shuttles in and out of the nucleus [39], but is enriched at sites of localised translation associated with paxillin in migrating fibroblasts [40]. Using human lung fibroblasts, we have demonstrated that ArpC2 mRNA associates with ribosomes during lamellipodia assembly and that levels of ArpC2 and Rac1 proteins increased at the leading edge of cells during spreading [41]. As DDX3X can bind to Rac1 mRNA and regulate protein levels, cell motility and metastasis [25], we have examined the functional relationships between DDX3X and mRNA-binding proteins during cell spreading. Using many complementary approaches, we now show that DDX3X can physically interact with PABP1 and with caprin-1, and co-localise at the leading edge of the cell. Furthermore, as depletion of DDX3X leads to decreased cell motility, these data provide a potential functional link between DDX3X, initiation factors and mRNA-binding proteins localised to the leading edge of migrating cells.

## Materials and methods

### Cell culture

MRC5 cells were routinely cultured in MEM (Minimal Essential Medium; Gibco) supplemented with 10% (v/v) foetal bovine serum (Labtech, U.K.) in a humidified atmosphere containing 5% CO_2_.

### Cell extracts

Cells were scraped into phosphate-buffered saline (PBS), pelleted by centrifugation in a cooled microcentrifuge at 10 000×***g*** at 4°C and resuspended in lysis buffer [20 mM MOPS (pH 7.4), 100 mM KCl, 1 mM DTT, 1 mM EDTA, 2 mM benzamidine, 25 mM NaF, 5 µg/ml leupeptin, 10 mM chymostatin, 1 µM microcystin LR and 1× EDTA-free protease inhibitor cocktail (Roche)]. After resuspension, cells were lysed by the addition of 0.5% (w/v) deoxycholate and 0.5% (v/v) Igepal followed by vortexing. Cell debris was removed by centrifugation in a cooled microcentrifuge at 10 000×***g*** at 4°C. For extraction of soluble protein prior to lysis, cells were incubated with digitonin buffer [42 µg/ml digitonin, 20 mM MOPS (pH 7.4), 0.2 mM EDTA, 150 mM NaCl, 2 mM DTT and 2 mM MgCl_2_] for 10 min at 4°C on a rotary platform; insoluble protein was removed by centrifugation in a cooled microcentrifuge at 10 000×***g*** at 4°C.

### Western blotting

Proteins were separated by SDS–PAGE, transferred to nitrocellulose membranes and visualised with the appropriate antibodies followed by HRP-conjugated secondary antibodies (DAKO, 1 : 2000). HRP activity was detected using the Pierce ECL Western Blotting Substrate followed by exposure to Amersham Hyperfilm ECL or using the Image Quant LAS 4000 imaging system. Antibody dilutions used for Western blotting were: anti-eIF4E [37] 1 : 10 000; anti-DDX3X (a gift from Dr R. Reed, Harvard Medical School, U.S.A.) 1 : 8000; anti-PABP1 (ab2160) 1 : 1000; anti-GAPDH (Ambion Am4300) 1 : 20 000; anti-Caprin-1 (Sigma HPA018126) 1 : 1000 and anti-HA (Cell Signaling) 1 : 2000.

### m^7^GTP-agarose affinity chromatography

Aliquots of extract containing 500 μg of protein were incubated with 50 µl of either γ-aminophenyl-m^7^GTP-Agarose C_10_-linked (Jena Biosciences) or Agarose beads alone (Sigma, U.K.) for 4 h at 4°C, with rotation. Recovered proteins were washed three times with lysis buffer supplemented with 0.1% (v/v) Igepal followed by one wash in PBS (downstream Western blotting) or three washes in PBS [downstream LC–MS (liquid chromatography–mass spectrometry) analysis]. Washed, bound proteins were recovered with either Laemelli buffer for Western blotting or urea buffer for LC–MS analysis [7 M urea and 2 M thiourea in 25 mM Tris–HCl (pH 7.5)].

### HA-DDX3X immunoprecipitation

Aliquots of extract containing 500 µg of protein were incubated with 50 µl of anti-HA-Agarose (Sigma, U.K.) for 2 h at 4°C, with rotation. Extracts were incubated in the presence or absence of 10 µg/ml RNAase1. Recovered proteins were washed three times with lysis buffer supplemented with 0.1% (v/v) Igepal, followed by one wash in PBS (downstream Western blotting) or three washes in PBS (downstream LC–MS analysis). Washed, bound proteins were recovered with either Laemelli buffer for Western Blotting or urea buffer for MS analysis [7 M urea and 2 M thiourea in 25 mM Tris–HCl (pH 7.5)].

### Immunoprecipitation

Aliquots of extract containing 500 μg of protein were incubated either with 20 µl of DDX3X (Sc-81247) antibody or 5 µl of PABP1 antibody (ab21060) for 2.5 h at 4°C with the addition of 50 µl of Protein G or Protein A-Sepharose for the last 30 min. Extracts were incubated in the presence or absence of 10 µg/ml RNAase1. Recovered proteins were washed three times with lysis buffer supplemented with 0.1% (v/v) Igepal followed by one wash in PBS (downstream Western blotting) or three washes in PBS (downstream LC–MS analysis). Washed, bound proteins were recovered with either Laemelli buffer for Western blotting or urea buffer for LC–MS analysis [7 M urea and 2 M thiourea in 25 mM Tris–HCl (pH 7.5)].

### Protein digestion with trypsin

Denatured protein was reduced by the addition of 1 mM DTT for 30 min at room temperature followed by alkylation with 55 mM iodoacetamide for 20 min at room temperature in the dark. For trypsin digestion, the sample was diluted to 1 M urea with 50 mM TEAB buffer and incubated at 37°C overnight with 0.2 µg of trypsin. The digestion was stopped by the addition of 3% (v/v) formic acid. Peptides were concentrated using a vacuum concentrator and purified on C18 spin columns (Pierce 89870) before resuspension for LC–MS.

### Dimethyl labelling for quantitative proteomics

Dimethyl labelling was performed exactly as described in Boersema et al. [42]. Samples were labelled with either formaldehyde or deuterated formaldehyde, producing a mass difference of 4 Da. For each dataset, one experimental sample was labelled with the light isotope and one experimental sample was labelled with the heavy isotope. Peptides were purified on C18 spin columns (Pierce 89870) before resuspension for LC–MS.

### CRISPR/Cas9 depletion of DDX3X

A type II CRISPR–Cas system was utilised as described [43]. The CRISPR sgRNA design tool was used to analyse a 1 kb fragment of the region of interest and locate suitable target sites. Both on- and off-target sites were computationally predicted, allowing selection of the three highest-ranked sgRNA sequences. Using Primer-Blast, oligonucleotides were designed to anneal ∼500 bp upstream and downstream from the intended target site in exon1 of DDX3X. A guide sequence RNA of 20 nucleotides was used to create a non-homologous end-joining (NHEJ) insertion/deletion event and a concomitant reading frame shift closely following the start codon of the DDX3X (ENSG00000215301) gene, using the double-nicking strategy with the Cas9 nickase mutant with paired guide RNAs to minimise off-target cleavage. Analysis of DDX3X mRNA levels by qRT-PCR showed at least 95% depletion when normalised to GADPH and HPRT1 (data not shown).

### Wound-healing assay

The Oris Universal Cell Migration Assembly kit was purchased from AMS Biotechnology (Europe) Ltd, and the assay was performed according to the manufacturer's instructions. Briefly, 5 × 10^4^ cells in 100 μl were loaded in triplicate into stopper-loaded wells in a 96-well plate. Cells were incubated in a humidified chamber (37°C, 5% CO_2_) for 4 h to permit cell attachment. To start cell migration, the stoppers were removed, cells were washed with sterile PBS and fresh DMEM was added. Images were taken at various indicated time points using an Optika XDS-2 light microscope (4× objective lens). Data were analysed with the ImageJ software (NIH, Bethesda, U.S.A.).

### Immunocytochemistry

Prior to fixation, MRC5 cells were seeded on collagen 1-coated coverslips to a density of 15 000 cells/cm^2^ and incubated for 45 min for cell spreading assays, or for 2–6 h for cell growing assays; fully confluent cells were obtained after 24 h of incubation. Processing of samples for immunofluorescence analysis by confocal microscopy, Z-sectioning and deconvolution analysis was as described previously [37,38,41]. Antibody dilutions used for imaging were: anti-eIF4E [37] 1 : 200; anti-DDX3X (a gift from Dr R. Reed, Harvard Medical School, U.S.A.) 1 : 500; anti-PABP1 (ab2160) 1 : 500; anti-caprin-1 (Sigma HPA018126) 1 : 500 and anti-FAK (Cell Signaling) 1 : 500. Actin was visualised with Alexa Fluor® 633 Phalloidin (A22284, Invitrogen) at 1 : 50 and nuclei with 12.5 ng/ml 4′,6′-diamidino-2-phenylindole hydrochloride (DAPI) for 5 min.

### Mass spectrometry, protein identification and quantification

Samples were analysed using an EASY-nLC 1000 liquid chromatography system coupled to a Q-Exactive mass spectrometer (LC–MS). The separation column and emitter was an *EASY-*Spray column, 50 cm × 75 µm ID; PepMap C18, 2 µm particles; 100 Å pore size. Buffer A was 2% (v/v) acetonitrile and 0.1% (v/v) formic acid, and buffer B was 100% (v/v) acetonitrile and 0.1% (v/v) formic acid. A gradient from 5 to 40% acetonitrile over 120 min was used to elute peptides for ionisation by electrospray ionisation and data-dependent MS/MS acquisition consisting of 1 full MS1 (*R* = 70 K) scan acquisition from 350 to 1500 *m/z* and 10 HCD type MS2 scans (*R* = 15 K). MS/MS charge targets were limited to 1 × 10^6^; the isolation window was set to 2.0 *m/z*; monoisotopic precursor selection, charge state screening and dynamic exclusion were enabled and charge states of +1, >4 and unassigned charge states were not subjected to MS2 fragmentation. Raw mass spectra were identified and quantified using Maxquant 1.5.15 using a 1% peptide and protein FDR. Searches were conducted against the uniprot SwissProt database. The database was supplemented with common contaminant proteins introduced during proteomic experiments. Searches were specified as tryptic with 1 missed cleavage, 7 ppm precursor ion mass tolerance, 0.05 Da fragment ion mass tolerance, fixed modifications of carbamidomethylation (C) and variable modification of oxidation (M), acetylation (N-term, Protein).

### Data deposition

The mass spectrometry proteomics data have been deposited to the ProteomeXchange Consortium via the PRIDE partner repository with the dataset identifier PXD006497 and data supporting this work are provided in Supplementary Data.

## Results and discussion

Whilst DDX3X has been shown to influence translation of specific mRNAs [3,4,9,25,26,35,44] and has a role in tumourigenesis [14,45], it is not known whether localised DDX3X protein has a role in translational control during cell spreading. Previously, we have shown that eIF4E, eIF4A, PABP1 and eIF4GI localise with nascent focal adhesions in spreading cells at the leading edge of migrating fibroblasts, in loci enriched with actively translating ribosomes [37,38,40]. DDX3X interacts with eIF4A, eIF4E, eIF4GI and PABP1 [3,26,44], and physically associates with G3BP-1, contributing to the formation of stress granules [26], sequestering specific mRNAs and halting their translation [27,28].

### A reduction in DDX3X levels impedes cell migration

**Figure 1.**
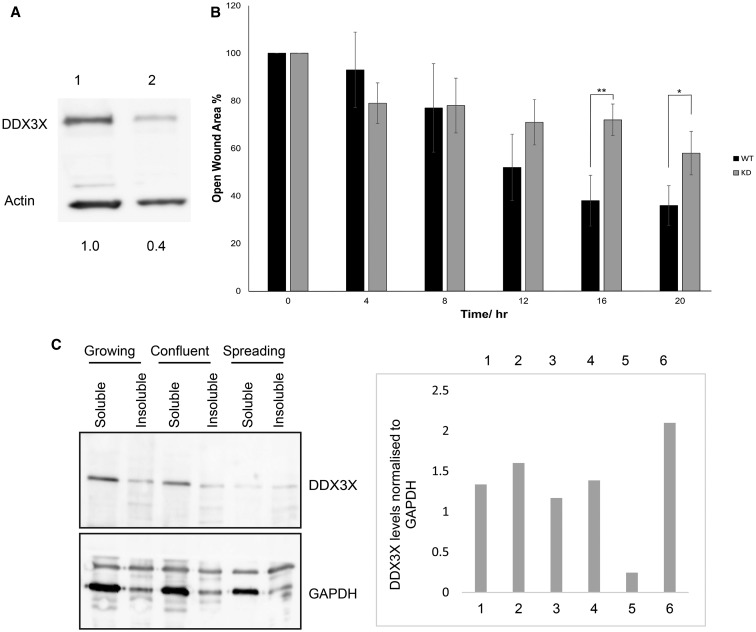
Reduction in DDX3X levels impedes cell migration. (**A**) Aliquots of extract (20 µg of total protein) from parental MRC5 (lane 1) or MRC5 cells partially depleted of DDX3X by CRISPR/Cas9 (lane 2) were resolved by SDS–PAGE and visualised using immunoblotting. Levels of DDX3X expression were quantified relative to actin (set at 1.0). (**B**) Wound-healing assays were carried out as described using parental (WT) and DDX3X-depleted (KD) cells over a period of 20 h. Following removal of the bung from confluent cultures, wound closure was measured using the Image J software and expressed as % of open wound area relative to time 0. Data are presented as the means ± SD, *n* = 3. All data were analysed using a one-way ANOVA test; *P ≤ 0.05; **P ≤ 0.01. (**C**) MRC5 cells were grown to either logarithmic or stationary growth phases, washed in PBS and incubated with digitonin to extract soluble protein, as described in the Materials and Methods section. For spreading cells, cells were harvested during logarithmic growth using trypsin and allowed to re-settle and spread on dishes until lamellipodia were established. Cells were scraped into lysis buffer and lysed to extract soluble and non-soluble protein. Upper panel: aliquots of protein (5 µg of total protein, ∼1/300 of the total soluble fraction and 1/50 of the insoluble fraction) were separated by SDS–PAGE and proteins were visualised by Western blotting. DDX3X levels were normalised to GAPDH (lower panel).

To examine whether there was an effect of DDX3X depletion on cell migration, we made several attempts to knockout expression using a CRISPR–Cas9 system in MRC5 fibroblasts [43]. Western blotting of a resulting cell clone showed that the surviving cells had only a partial knockdown of DDX3X to ∼40% of control levels (Figure 1A), although mRNA levels were severely depleted (data not shown). This is unlikely to be due to the heterogeneity of the selected clone of cells and was seen is five separate surviving clones of cells (data not shown). As DDX3X modulates efficient expression of Rac1 to regulate actin dynamics [24], contributing to cell adhesion and motility [25], we analysed Rac 1 levels following partial knockdown of DDX3X. In these cells, the reduction in DDX3X protein levels did not reduce the steady-state Rac1 protein level in spreading cells (data not shown). This could reflect the incomplete knockdown; however, at this time, we cannot discount effects of partial knockdown of DDX3X on localised translation of Rac1. To examine the effect of partial depletion of DDX3X on cell migration, we monitored wound closure in serum-starved cells (to reduce cell proliferation) using stopper-loaded culture dishes. As shown in Figure 1B, partial depletion of DDX3X resulted in a decreased rate of wound closure. As there was no effect of partial depletion of DDX3X on cell viability or proliferatrion rates (data not shown), these data suggest a role for DDX3X in cell spreading and migration.

To begin to understand this role of DDX3X, soluble cytosolic proteins and insoluble proteins were separated from MRC5 fibroblasts during logarithmic (growing) or stationary (confluent) growth phases, and from cells in early phases of spreading when laemellopodia were first formed [38]. Soluble proteins were extracted using digitonin, and both soluble and non-soluble protein fractions were separated by SDS–PAGE and DDX3X was visualised by immunoblotting. DDX3X expression levels were quantified by normalisation to the cytosolic marker, GAPDH. Figure 1C shows that there was a reduction in DDX3X protein levels in the soluble fraction of spreading cells when compared with those in the soluble fraction of either confluent or growing cells; the amount of DDX3X extracted from insoluble fractions remained constant although the total level of DDX3X extracted was reduced relative to growing cells. This might reflect relocalisation of a population of DDX3X to the nucleus (see Supplementary Figure S1B) , which was not lysed under these conditions. Overall, these data suggest the maintenance of DDX3X in insoluble membrane fractions compared with soluble cytosolic protein on cell spreading.

### The DDX3X interactome

**Figure 2.**
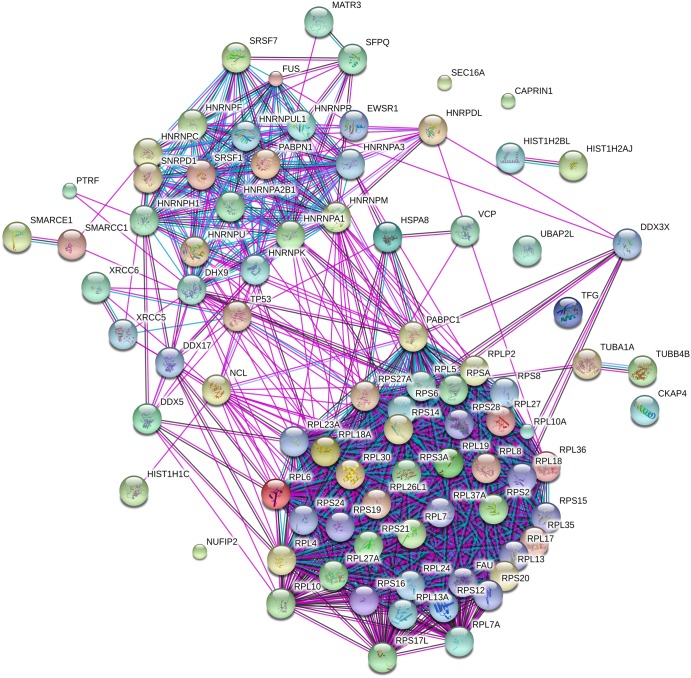
LC–MS analysis of the DDX3X interactome. HA-DDX3X and associated protein were immunoprecipitated from total cell extracts prepared from spreading cells, digested with trypsin and analysed by LC–MS, as described in the Materials and Methods section. A PPI network of these data was generated using STRING [46], reporting a network showing connections for the uploaded proteins.

As DDX3X has been reported to interact with many proteins, we have used co-immunoprecipitation coupled with LC–MS to investigate binding partners of DDX3X during cell spreading. DDX3X immunoprecipitates were prepared from both spreading MRC5 cells using an antibody to the endogenous protein and from spreading MRC5 cells expressing HA-tagged DDX3X; resin-only controls were included for both experiments. Recovered proteins were digested with trypsin, differentially labelled with formaldehyde and analysed by LC–MS, as described in the Materials and Methods section (Supplementary Datatset S1). To identify proteins co-isolating with DDX3X, proteins which were enriched two-fold or more in both experiments were selected as interactors and mean ratios were calculated (Supplementary Datatset S2). A protein–protein interaction (PPI) network of these data (DDX3X interactome) was generated using the Search Tool for the Retrieval of Interacting Genes/Proteins (STRING) database [46]. The STRING analysis tool quantitatively integrates interaction data from high-throughput experiments, genomic context, co-expression and other literature. STRING reports a network showing connections for the uploaded proteins (Figure 2) with functional enrichments for this network identified in Supplementary Datatset S3. These data show that the biological processes with the highest scoring functional enrichments were for multiple proteins associated with mRNA metabolism, including translation initiation (GO:0006413) and viral transcription (GO:0019083).

### The DDX3X interactome includes caprin-1 and PABP1

These data did not show an interaction between DDX3X and eIF4E; however, PABP1, which has also been reported to interact with DDX3X [3,26,44], was confirmed as a binding partner of DDX3X in spreading cells. Further analysis of the DDX3X interactome shown in Supplementary Datatset S2 identified caprin-1 as a co-precipitating protein [30–32]. Caprin-1 has been shown to localise to the leading edge of cells, selectively bind mRNA [33] and to interact directly with G3BP-1 to promote stress granule formation [34]. Overexpression of caprin-1 promotes the proliferation and invasion of breast cancer cells [32] and is involved in the development of osteosarcoma [47].

**Figure 3.**
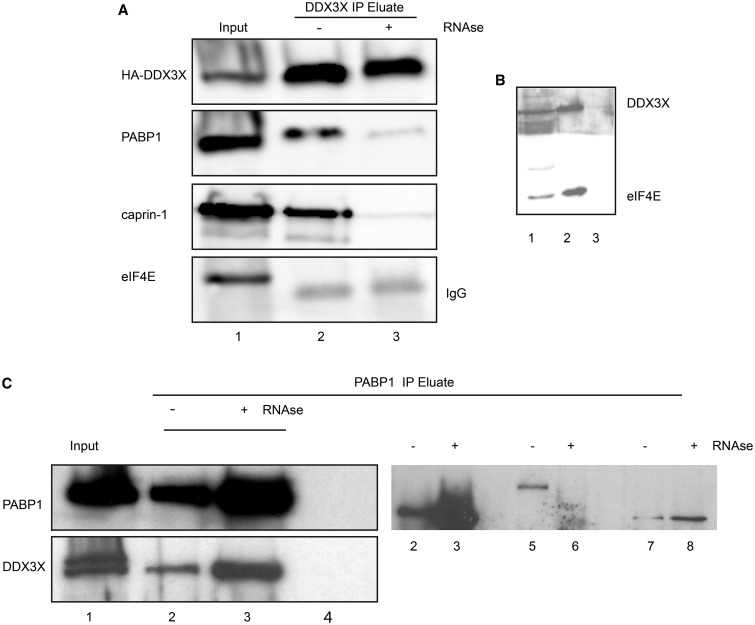
Interaction of HA-DDX3X with PABP1 or caprin-1 is RNA-dependent. (**A**) Cell lysates were prepared from spreading cells expressing HA-DDX3X; aliquots containing 500 µg of total protein were incubated with anti-HA-Agarose resin, in the presence or absence of RNAse1, to recover HA-DDX3X and associated proteins. Total extract (5% of input; lane 1) or recovered proteins (lanes 2 and 3) were separated by SDS–PAGE and visualised using immunoblotting using the antisera shown. (**B**) The soluble fraction of confluent cells (lane 1; 5% input) was incubated with m^7^GTP-Agarose to isolate eIF4E and associated proteins (lane 2), or with Agarose resin alone (lane 3). Protiens which co-isolated with eIF4E were separated by SDS–PAGE and visualised using immunoblotting. (**C**) Cell lysates were prepared from spreading cells; aliquots containing 500 µg of total protein were incubated with antibody specific to PABP1 followed by Protein A-Sepharose to recover PABP1 and associated proteins. Lysates were incubated with or without antibody, in the presence or absence of RNAse1, as described in the Materials and Methods section. Total protein and recovered proteins were separated by SDS–PAGE and visualised using immunoblotting using the antisera shown. Total extract (5% of input; lane 1) recovered proteins (lanes 2–4) and incubated with antibody (lanes 2 and 3) and resin only (lane 4).

To address whether DDX3X co-isolates with PABP1 or caprin-1 in spreading cells, HA-DDX3X immunoprecipitates were separated by SDS–PAGE and co-precipitating proteins were visualised using immunoblotting. Figure 3A shows that both PABP1 and caprin-1 were recovered with HA-DDX3X from spreading cells. The addition of RNAse1 to the immunoprecipitation incubation buffer greatly reduced the interaction between DDX3X and both PABP1 and caprin-1, suggesting that the majority of protein interaction observed is RNA-dependent. DDX3X was also identified in PABP1 immunoprecipitates (Figure 3A). In contrast, and consistent with our LC–MS data, eIF4E did not co-purify with HA-DDX3X under these conditions.

### The mRNA cap-binding protein interactome includes DDX3X and proteins involved in RNA metabolism

Previous reports have identified interactions between DDX3X, eIF4E and many initiation factors [3,26,35]. Since eIF4E was not identified as part of the DDX3X interactome (Figure 2), the association between DDX3X and eIF4E was further investigated in spreading cells using m^7^GTP-Agarose to isolate eIF4E and associated proteins. Proteins which co-isolated with eIF4E were separated by SDS–PAGE, and DDX3X recovery was visualised using immunoblotting. Figure 3B shows that a population of DDX3X co-isolated with eIF4E and was not retained on the Agarose resin alone. To further confirm this, cap-binding proteins from spreading cells were digested with trypsin and differentially labelled with formaldehyde and analysed by LC–MS (Supplementary Datatset S4). To identify proteins co-isolating with eIF4E, proteins which were enriched two-fold or more on m^7^GTP-Agarose, in two replicates, were selected and mean protein ratios were calculated (Supplementary Datatset S5). The experiment was repeated extracting eIF4E from confluent cells. Both data sets confirmed a functional interaction between DDX3X and eIF4E. As caprin-1 was not identified as part of the eIF4E interactome, it is likely that DDX3X is present in multiple interactomes within the cell. As recovery of DDX3X on m^7^GTP-Agarose was insensitive to nuclease (data not shown) and eIF4E was not recovered in DDX3X immunoprecipitates (Figure 3A and Supplementary Datatset S2), these data suggest an indirect interaction with eIF4E, possibly via eIF3 [35], PABP1 [26] or eIF4GI [3].

### DDX3X is closely associated with caprin-1 and PABP1 in migrating MRC5 fibroblasts

**Figure 4.**
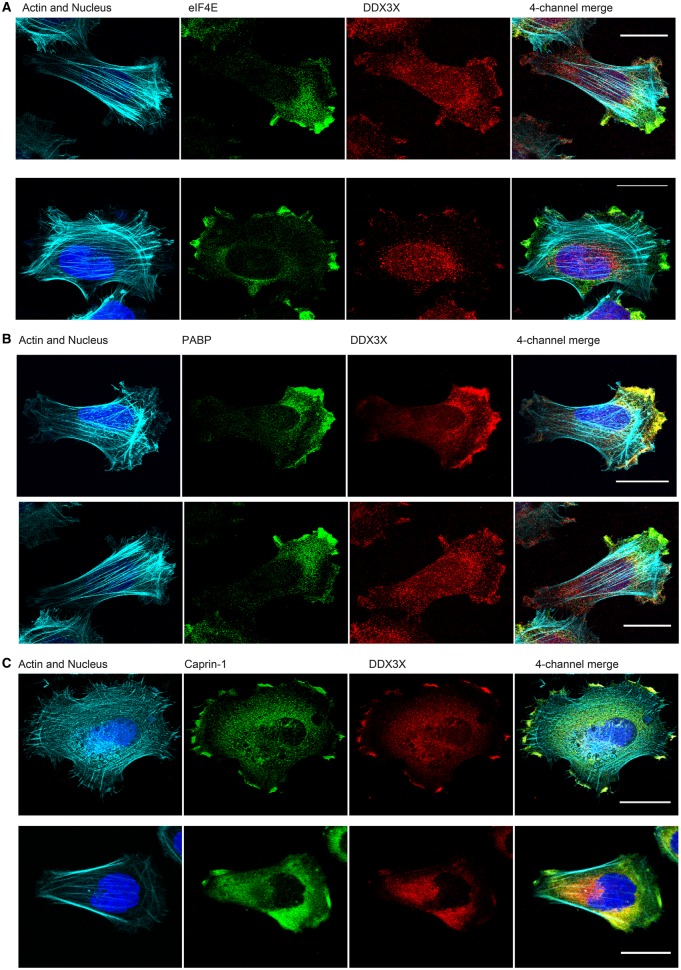
DDX3X is closely associated with caprin-1 and PABP1 at the leading edge of migrating MRC5 fibroblasts. (**A**) MRC5 cells were seeded on collagen 1-coated coverslips to a density of 15 000 cells/cm^2^ and incubated for 45 min to allow for formation of lamellipodia. Processing of samples for immunofluorescence analysis by confocal microscopy, Z-sectioning and deconvolution analysis was as described in the Materials and Methods section. In addition to detection of the nucleus and actin cytoskeleton, staining was carried out for eIF4E and endogenous DDX3X. Scale bar is 20 µm. (**B**) MRC5 cells were seeded, processed and analysed as described in (**A**). In addition to detection of the nucleus and actin cytoskeleton, staining was carried out for PABP1 and endogenous DDX3X. Scale bar is 20 µm. (**C**) MRC5 cells were seeded, processed and analysed as described in (**A**). In addition to detection of the nucleus and actin cytoskeleton, staining was carried out for caprin-1 and endogenous DDX3X. Scale bar is 20 µm.

As caprin-1 [33], eIF4E [37,38] and PABP1 [40,48] can all be localised to the leading edge of cells, we examined whether they are co-localised with DDX3X in spreading cells. Cells were seeded onto collagen-covered coverslips and allowed to spread until lamellopodia were visualised. Confocal microscopy/immunofluorescence was used to visualise the proteins; staining for paxillin and focal adhesion kinase (FAK) was included as markers of focal adhesions [40]. Figure 4A demonstrates that a population of DDX3X can be seen at the leading edge of the cell; although eIF4E is also found in this compartment, there was a relatively low level of co-localisation with DDX3X. As predicted, both PABP1 (Figure 4B) and caprin-1 (Figure 4C and Supplementary Figure S1A) were also found at the leading edge of the cells, co-localised with DDX3X. Using Leptomycin B to maintain a large proportion of DDX3X in the nucleus did not affect the localisation of PABP1 to the periphery of spreading cells. These data suggest that the localisation of PABP1 is largely independent of DDX3X (Supplementary Figure S1B). In contrast with caprin-1, there was little obvious co-localisation of DDX3X with paxillin (Supplementary Figure S2A) or FAK (Supplementary Figure S2B) under these conditions.

Finally, we have compared the interactomes of endogenous DDX3X and HA-DDX3X recovered from spreading cells using LC–MS and compared the functional annotation of proteins recovered in both data sets (Supplementary Datatset S6). These data show that the top functional annotations found in both data sets map onto known functions of DDX3X in mRNA metabolism, translation process and regulation, indicating that tagged DDX3X expressed at this level was likely functioning in a similar manner to the endogenous protein.

In conclusion, our data suggests that DDX3X is required at the leading edge of the cell for efficient spreading on collagen. A population of this protein is clearly associated with PABP1 and caprin-1, findings supported by LC–MS studies (Figure 2 and Supplementary Datatsets S1 and S2) and consistent with reported findings [40]. As the majority of the interaction between DDX3X, PABP1 and caprin-1 is RNA-dependent (Figure 3A), these data suggest that DDX3X could be regulating the translation of specific subsets of mRNA with complex 5′-UTR structures at the leading edge of the cell. Inhibition of translation initiation or elongation in actively growing cells did not prevent the co-precipitation of DDX3X and PABP1 (data not shown). A similar role has been suggested for the RNA-binding protein, Larp1; it can also be found in this compartment, interacting with mRNA, PABP1, eIF4E, ribosomal subunits and the cytoskeleton [49]. As DDX3X can bind to Rac1 mRNA and regulate protein levels, cell motility and metastasis [25] and levels of ArpC2 and Rac1 proteins increased at the leading edge of cells during spreading [41], it is likely that localised DDX3X plays a key role in specific translation here. Further work is required to understand the functional relationships between DDX3X protein domains, localisation of the protein and the identity of specific mRNAs regulated in this cellular compartment.

## Abbreviations

5′-UTR: 5′-untranslated region
FAK: focal adhesion kinase
G3BP-1: RasGap SH3 domain-binding protein-1
LC–MS: liquid chromatography–mass spectrometry
PABP1: poly(A)-binding protein 1
PBS: phosphate-buffered saline
PPI: protein–protein interaction
STRING: Search Tool for the Retrieval of Interacting Genes/Proteins

## Supplementary Material

Supplementary Figures

Supplementary Datatset S1

Supplementary Datatset S2

Supplementary Datatset S3

Supplementary Datatset S4

Supplementary Datatset S5

Supplementary Datatset S6
