# Supplementary material for: The helicase, DDX3X, interacts with poly(A)-binding protein 1 (PABP1) and caprin-1 at the leading edge of migrating fibroblasts and is required for efficient cell spreading
Source: Biochem J. 2017 Aug 31;474(18):3109–20. doi: 10.1042/BCJ20170354 (PMC5577505; doi:10.1042/BCJ20170354)
Supplement: Supplementary Figures [file BCJ-474-3109-s1.pdf]

Legends to Supplementary Figures and Datasets

Figures

Supplementary Figure S1. *DDX3X is closely associated with caprin-1 and PABP1 at the leading edge of migrating MRC5 fibroblasts*

Panel A. MRC5 cells were seeded, processed and analysed as described in Fig.4. In addition to detection of the nucleus and actin cytoskeleton, staining was carried out for caprin-1 and endogenous DDX3X. Scale bar is 20µm. **Panel B.** MRC5 cells were pretreated with 5 µM Leptomycin B for 3 hours, trypsinised and seeded on collagen 1-coated coverslips at 15000 cells/cm^2, and incubated for 45 minutes to allow for formation of lamellipodia. Processing of samples for immunofluorescence analysis of PABP1 and endogenous DDX3X by confocal microscopy was as described in Fig.4. Scale bar is 20µm.

Supplementary Figure S2. *DDX3X does not co-localise with paxillin or FAK at the leading edge of the cell*

MRC5 cells were seeded on collagen 1-coated coverslips at 15000 cells/cm^2, and incubated for 45 minutes to allow for formation of lamellipodia. Processing of samples was as described in Supplementary Figure S1. In addition to detection of the nucleus and actin cytoskeleton, staining was carried out for paxillin, FAK and endogenous DDX3X, as indicated. Scale bar is 20µm.

Dataset

Supplementary Datatset S1*. Proteins co-precipitating with DDX3X*

DDX3X immunoprecipitates were digested with trypsin, differentially labelled with formaldehyde and analysed by LC-MS, as described in the Materials and Methods. Sequences were searched against a human SwissProt database and matching peaks quantified using a MaxQuant algorithm, as described.

Supplementary Datatset S2. *Enrichment ratios for proteins co-precipitating with DDX3X*

Using Perseus software (http://www.perseus-framework.org), proteins which were enriched two-fold or above in two replicates were combined and the mean value calculated. The Excel data sheet shows mean ratios for proteins enriched 2-fold or above.

Supplementary Datatset S3. ***Functional enrichments for the PPI network of DDX3X co-precipitating proteins***

The PPI network was generated using STRING database. Proteins enriched two-fold or above in HA-DDX3X immunoprecipitates (Supplementary Datatset S2) were uploaded into the database. Interactions determined experimentally, from co-expression studies and from database searches were included in the search with a minimum interaction score of 0.4. The Excel sheet shows functional enrichments for GO terms designated as Biological .

Supplementary Datatset S4 ***Proteins co-precipitating with eIF4E on m^{7}GTP-Agarose***

Eluates from m^{7}GTP-Agarose were digested with trypsin, differentially labelled with formaldehyde and analysed by LC-MS, as described. Sequences were searched against a human SwissProt database and matching peaks quantified using a MaxQuant algorithm as described in matrials and methods.

Supplementary Datatset S5. ***Enrichment ratios for proteins co-precipitating with eIF4E***

Using Perseus software proteins which were enriched two-fold or above in two replicates were combined and the mean value calculated. The Excel data sheet shows mean ratios for proteins enriched 2-fold or above.

Supplementary Datatset ***S6. Functional annotation of proteins enriched with endogenous and HA-DDX3X co-precipitating proteins***

DDX3X and HA-DDX3X and associated protein was immunoprecipitated from total cell extracts prepared from spreading cells, digested with trypsin and analysed by LC-MS, as described in the Materials and Methods. The Excel sheet shows functional enrichments for GO terms designated as Biological which were observed in both data sets.

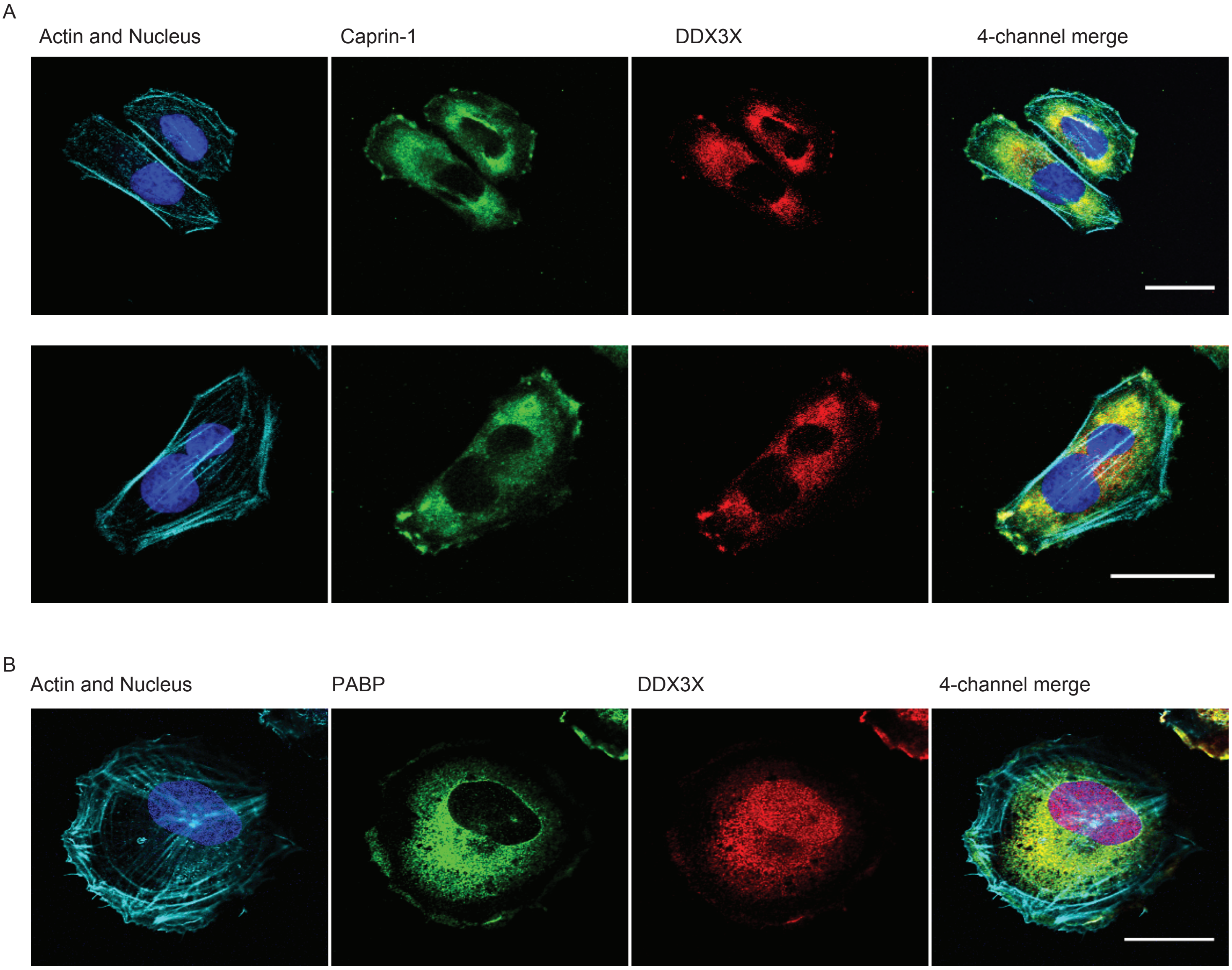
A
Actin and Nucleus
Caprin-1
DDX3X
4-channel merge
B
Actin and Nucleus
PABP
DDX3X
4-channel merge

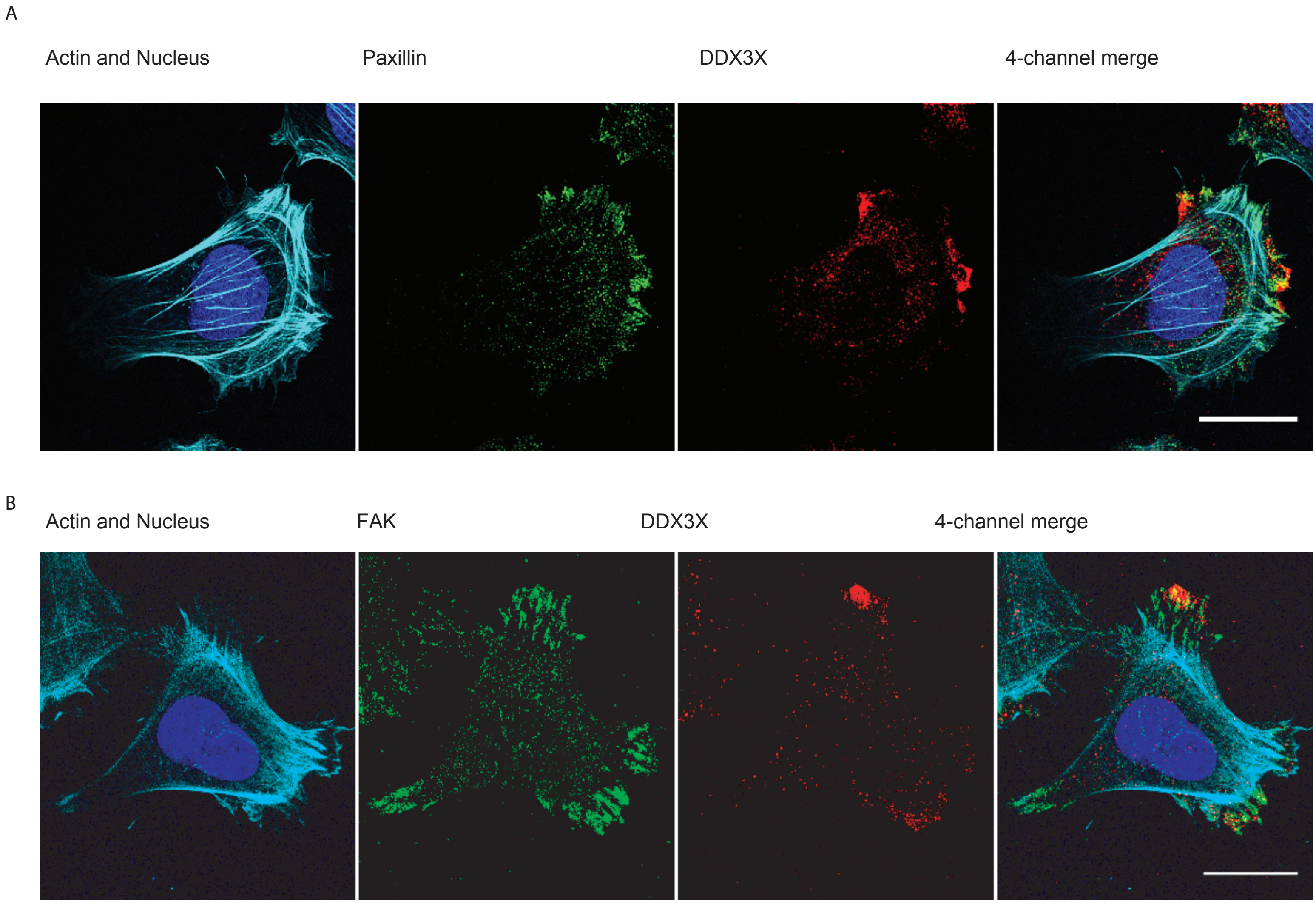
A
Actin and Nucleus
Paxillin
DDX3X
4-channel merge
B
Actin and Nucleus
FAK
DDX3X
4-channel merge
